# The Functions and Mechanisms of Tendon Stem/Progenitor Cells in Tendon Healing

**DOI:** 10.1155/2023/1258024

**Published:** 2023-09-12

**Authors:** Jingwei Lu, Hui Chen, Kexin Lyu, Li Jiang, Yixuan Chen, Longhai Long, Xiaoqiang Wang, Houyin Shi, Sen Li

**Affiliations:** ^1^School of Physical Education, Southwest Medical University, Luzhou, China; ^2^Geriatric Department, The Affiliated Traditional Chinese Medicine Hospital of Southwest Medical University, Luzhou, China; ^3^Spinal Surgery Department, The Affiliated Traditional Chinese Medicine Hospital of Southwest Medical University, Luzhou, China; ^4^Division of Spine Surgery, Department of Orthopedic Surgery, Nanjing Drum Tower Hospital, Affiliated Hospital of Medical School, Nanjing University, Nanjing, China

## Abstract

Tendon injury is one of the prevalent disorders of the musculoskeletal system in orthopedics and is characterized by pain and limitation of joint function. Due to the difficulty of spontaneous tendon healing, and the scar tissue and low mechanical properties that usually develops after healing. Therefore, the healing of tendon injury remains a clinical challenge. Although there are a multitude of approaches to treating tendon injury, the therapeutic effects have not been satisfactory to date. Recent studies have shown that stem cell therapy has a facilitative effect on tendon healing. In particular, tendon stem/progenitor cells (TSPCs), a type of stem cell from tendon tissue, play an important role not only in tendon development and tendon homeostasis, but also in tendon healing. Compared to other stem cells, TSPCs have the potential to spontaneously differentiate into tenocytes and express higher levels of tendon-related genes. TSPCs promote tendon healing by three mechanisms: modulating the inflammatory response, promoting tenocyte proliferation, and accelerating collagen production and balancing extracellular matrix remodeling. However, current investigations have shown that TSPCs also have a negative effect on tendon healing. For example, misdifferentiation of TSPCs leads to a “failed healing response,” which in turn leads to the development of chronic tendon injury (tendinopathy). The focus of this paper is to describe the characteristics of TSPCs and tenocytes, to demonstrate the roles of TSPCs in tendon healing, while discussing the approaches used to culture and differentiate TSPCs. In addition, the limitations of TSPCs in clinical application and their potential therapeutic strategies are elucidated.

## 1. Introduction

Tendon plays an essential role in joint stability and is composed mainly of collagen fibers and tendon-resident cells [1]. Along with the economic development and the popularity of sports activities, tendon injury is increasing annually. According to statistics, the incidence of lower limb tendinopathy is 10.52 per 1,000 person-years [2]. Tendinopathy (chronic tendon injury) is a widespread clinical problem in orthopedics, with pain and dysfunction as its typical symptoms, and its main pathological changes are collagen fiber disorders and vascular increase [3, 4]. The etiology of tendinopathy is complex and can be broadly divided into intrinsic and extrinsic factors. Intrinsic factors include age, genetics, systemic diseases, diabetes, biomechanics, and so forth. Extrinsic factors include physical load, environment, occupation, and so forth [5–7]. Since tendinopathy is highly prevalent but poorly curable, it not only impairs individuals' quality of life but also increases society's financial burden [8].

Management of tendon injury can be divided into conservative treatments, including NSAIDs, steroid injections, eccentric exercise, platelet rich plasma injections, and so forth. Surgical treatment is used when conventional medicine fails, or the tendon ruptures [9]. However, these treatments for tendinopathy are not optimal as they are mostly intended to relieve pain and reduce inflammation and do not improve the structural function or biomechanical properties of the healing tendon [10]. In recent years, stem cell therapy has received widespread attention [11]. Tendon stem/progenitor cells (TSPCs) have the potential to differentiate into tenocytes spontaneously. Besides, TSPCs have a higher proliferation capacity and a stronger differentiation potential [12]. Therefore, compared with bone marrow mesenchymal stem cells (BMSCs) and adipose-derived stem cells (AMSCs), TSPCs are superior in tendon regeneration [12].

TSPCs are a unique cell population recently discovered in the human and rat tendons, primarily in an extracellular matrix (ECM) composed of biglycan (Bgn) and fibromodulin (Fmod) [13]. TSPCs, like other stem cells, have the potential for self-renewal and multidirectional differentiation [14]. Differently from other stem cells, TSPCs express higher levels of tendon-related genes (e.g., Scx, Tnmd) [13]. Recently investigators have examined the effects of TSPCs on the process of tendon repair. The investigators found that TSPCs therapy significantly accelerated tendon healing and also discovered that TSPCs had an effect in all three phases of tendon repair, in addition to increasing the ultimate strength of the repaired tendon [15–18].

The role and mechanisms of TSPCs in tendon repair have been demonstrated in several studies; however, the detailed mechanisms have not been elucidated. Some studies have proved that TSPCs impede tendon healing and consequently result in tendinopathy. Therefore, the purpose of this article is to summarize the role and mechanisms of TSPCs in the three stages of tendon healing. Similarly, the negative effects of TSPCs on tendon healing as a critical factor in the pathogenesis of chronic tendon injury (tendinopathy) are discussed. In addition to this, the approaches used to culture and differentiate TSPCs are discussed, as well as the limitations of the clinical application of TSPCs and potential therapeutic approaches.

## 2. Tendon Stem/Progenitor Cells are Distinguished from Tenocytes and Other Mesenchymal Stem Cells

### 2.1. Comparison of Tenocytes and Tendon Stem/Progenitor Cells

Tenocytes and TSPCs are the principal cell types in tendon tissue. The tenocytes are a special type of fibroblast that makes up approximately 95% of the tendon tissue [19]. Mature tenocytes are spindle-shaped and have a bulge around the cell. The tenocyte is located between collagen fibers and it is responsible for the production of ECM including the secretion of collagen [20]. In fact, the specific markers of tenocytes are uncertain, however, the identification of tenocytes is usually determined by tendon differentiation markers, including Scx, Tnmd, and type I collagen (colⅠ) [21]. The expression of tenascin-C, thrombospondin-4, and tenomodulin, markers of tendon development, is higher in tenocytes than in TSPCs [22]. The proliferation and migration of tenocytes play a crucial role in the healing of tendon injury.

TSPCs are distinctive cell populations with self-renewing, clonal, and multidirectional differentiation potential, which were originally identified in mouse patellar tendon tissue by Bi et al. [13]. TSPCs are located in a niche composed mainly of ECM, which consists mainly of two small proteoglycans, Bgn and Fmod. Subsequently, TSPCs from different sites (patellar tendon, Achilles tendon, supraspinatus tendon, and so forth) of rat, rabbit, and pig have been extracted by various researchers [23–25]. TSPCs from diverse sites of different species have different cell morphologies, including pebble-shaped, spindle-shaped, and rounded, and some are similar to tenocytes in morphology [25, 26]. Compared to tenocytes, it is smaller in size, has a larger nucleus, and it proliferates more rapidly than tenocytes [25]. TSPCs do not have specific markers, similar to other stem cells, Oct-4, SSEA-4, and nucleostemin, as its marker genes, and CD44, CD90, and CD105 as its surface markers [27]. However, unlike other stem cells, all TSPCs express colⅠ, and they express higher levels of tendon-related genes including Scx, Tnmd, and tenascin-C [13].

In summary, TSPCs and tenocytes are distinguished from each other in terms of cell morphology, differentiation potential, and cell markers. In addition to this, TSPCs form dense, close colonies, whereas tenocytes form large, sparse colonies [22]. Since fewer experiments have compared TSPCs and tenocytes in the same species, this paper presents a summary and compares them based on the available experiments (Table 1).

### 2.2. Comparison of Tendon Stem/Progenitor Cells and Other Mesenchymal Stem Cells

Mesenchymal stem cells (MSCs) are stromal cells that have the ability to self-renew and exhibit multispectral differentiation [28]. MSCs have an important role in tissue repair. Equally, MSCs have great potential for use in the treatment of tendinopathy. The following section highlights the differences between TSPCs and BMSCs and AMSCs.

BMSCs are usually obtained from the iliac crest by minimally invasive puncture and then isolated by density centrifugation [13]. BMSCs are clonogenic, self-renewing, and differentiate into osteoblasts and tenocytes, and are relatively widely used in tissue engineering [29]. However, it has been reported that BMSCs show a remarkable decline in number and proliferative capacity with age [30].

The population of stem progenitor cells isolated from adipose is called AMSCs, which also have stem cell properties [31]. AMSCs have the advantages of being widely available and easy to obtain, and likewise, they have the ability to differentiate in multiple directions. In addition, AMSCs have an excellent advantage in ECM remodeling [32].

TSPCs are a type of MSCs with the ability of clonogenicity, self-renewal, and multipotent differentiation, which is a population of progenitor cells identified from tendon tissue by Bi et al. [13]. TSPCs not only have stem cell properties, but they also highly express tendon-related genes such as Scx, Tnmd, and Comp, which is an advantage not possessed by the previous two types of MSCs. Compared with BMSCs, TSPCs can express more Otc4, which has a strong proliferation and cloning ability [12]. Moreover, in the treatment of patellar tendon injury in rats, although both TSPCs and BMSCs can promote tendon repair, TSPCs are more suitable for tendon regeneration in vivo than BMSCs [33].

TSPCs have the same stem cell properties (cloning, self-renewal, multidirectional differentiation) as the other two types of MSCs, and both of their exosomes can promote tendon healing. Unlike the other two types of MSCs, TSPCs highly express tendon-related genes Scx and Tnmd and have the potential to spontaneously differentiate into tendons. In conclusion, TSPCs have higher proliferation potential, form more cell colonies, and express more tendon markers. In addition to this, TSPCs contribute to the synthesis of collagen types I and III [34]. Therefore, TSPCs can be used as ideal cells in tendon repair.

## 3. Subpopulations of TSPCs

### 3.1. Regional Differences in Stem Cell/Progenitor Cell Populations

Stem/progenitor cell populations are available not only in the tendon proper but also in the peritendon, with different properties existing in various regions of the progenitor cell pool [35–37]. However, both progenitor cells from the tendon proper and the peritendon are pluripotent and have a certain similarity in marker expression. For instance, the majority of clonable cells isolated from the tendon proper and peritendon showed reactivity to the fibroblast markers Cd90.2 and Cd44. However, progenitor cells from the tendon proper and the peritendon are also region specific [35]. First, cells from the tendon proper had more progenitor colonies than those from the peritendon. Also, a higher percentage of clonal progenitor cells from the tendon proper were positive for Sca-1 than those from the peritendon [35]. Second, the expression levels of tenomodulin (Tnmd) and scleraxis (Scx) were significantly increased in cells from the tendon proper compared to the peritendon cells, indicating enrichment of stem/progenitor cells of tendon origin [35]. Finally, there is a relatively increased vascular (endomyosin) and pericyte (Cd133) marker in the peritendon cells compared to the cells from the tendon proper. In addition to this, the potential for differentiation of peritendinous cells into myofibroblasts was observed to be higher [35, 36].

In conclusion, both the tendon proper and the peritendinous cell population can be multidifferentiated, and both express stem cell markers. The differing feature is that isolated stem/progenitor cells from within the tendon express higher levels of tendon markers, while peritendinous progenitors express higher levels of pericyte and vascular markers [37]. Furthermore, peritendinous cells migrate faster, duplicate more rapidly, and have a higher potential to differentiate into myofibroblasts.

### 3.2. Potential New Sources of Subpopulations of TSPCs and Their Identification

The perivascular wall may be a potential source of a subpopulation of TSPCs [38]. A majority of TSPCs originate from the tendon itself, but many investigations have shown that TSPCs are also present in the epitenon, which is mostly derived from pericytes or perivascular cells of the vascular system [39]. For example, Tan et al. used the iododeoxyuridine labeling retention method for labeling stem cells in rat patellar tendons both with and without injury. Colocalization of labeled retained cells (LRC) with different markers was accomplished by immunofluorescence staining. They found a proportion of LRCs within the vessel wall and found that some LRCs in the window wound expressed CD146. This suggests that a proportion of TSPCs are derived from the vessel wall [40].

Endomucin (Emcn), Musashi1 (Msi1), and Cd133, CD146 could be used as markers of vascular TSPCs. In addition, Yin et al. [41] identified a subpopulation of nestin^+^ TSPC in the tenocyte population by single-cell analysis. It has been shown that nestin is highly expressed in human Achilles tendon TSPCs and that it is mainly distributed in the perivascular region, suggesting that nestin may be a candidate marker for vascular-derived TSPCs.

## 4. The Mechanisms of Tendon Stem/Progenitor Cells in Tendon Healing

**Figure 1:**
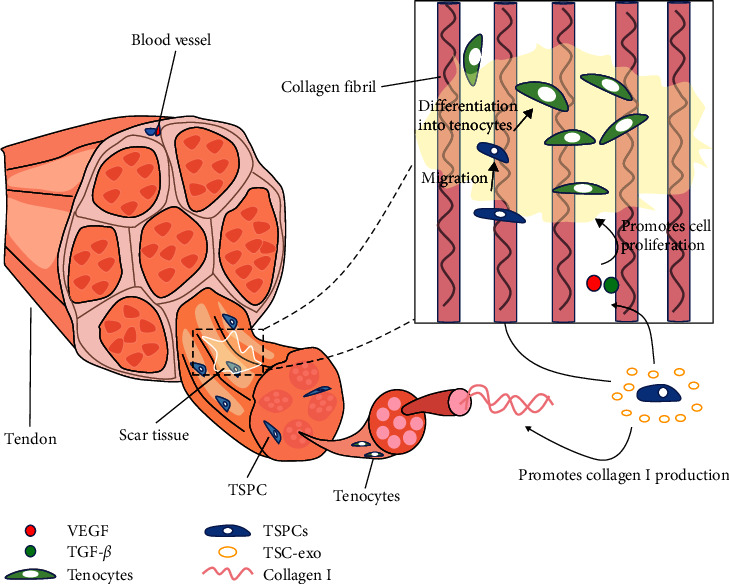
Mechanisms of the role of TSPCs in the process of tendon healing. TSPCs promote cell proliferation and migration, as well as collagen synthesis. TSPCs, tendon stem/progenitor cells; TSC-Exo, tendon stem cell-derived exosomes; VEGF, vascular endothelial growth factor; TGF-*β*, transforming growth factor beta.

The healing of tendon tissue, like skin tissue, can be divided into three phases: the inflammatory phase, the cells proliferation phase, and the cells remodeling phase [42, 43]. It has been shown that TSPCs can promote tendon healing [42]. The inflammatory phase occurs within 1 week of tendon injury, when vascular permeability increases and a large number of inflammatory cells move into the healing site. This also stimulates the production of a large number of growth factors and cytokines, when TSPCs are also activated and work together at the site of injury. During the proliferation and remodeling phase, tenocytes proliferate and deposit themselves at the site of injury [44]. Type III collagen (colIII) is thought to be essential in the early stages of tendon repair, laying the foundation for the subsequent production of colⅠ. TSPCs promote collagen production and allow collagen fibers to be ordered [45]. Many investigations have demonstrated the positive effects of TSPCs in the three stages of tendon healing and their structural molecular mechanisms on tendons are shown in Figure 1.

### 4.1. Tendon Stem/Progenitor Cells Modulate the Inflammatory Process

**Figure 2:**
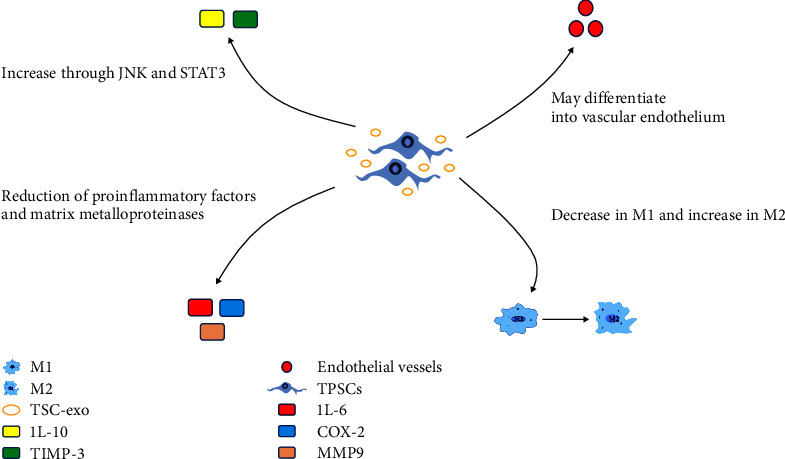
The role of TSPCs in the inflammatory phase. TSPCs, tendon stem/progenitor cells; TSC-Exo, tendon stem cell-derived exosomes; M1, macrophage 1; M2, macrophage2; IL-10, interleukin-10; IL-6, interleukin-6; MMP-9, matrix metalloproteinase-9.

The inflammatory phase is the initial period of tendon healing, which involves inflammatory response and angiogenic processes [46, 47]. Although the inflammatory response is an important part of tendon healing, either a disrupted or excessive inflammatory response can prevent tendon healing and excessive inflammation can cause scars to form at the injury site [48]. Many researchers have shown that TSPCs play an essential role in tendon injury healing, not only by modulating the inflammatory response, but also by inhibiting the formation of scarring and fibrosis [16, 17]. The detailed mechanisms by which TSPCs regulate the inflammatory response are shown in Figure 2. In addition to this, studies have demonstrated that TSPCs can inhibit the proliferation of lymphocytes [49]. TSPCs inhibit the inflammatory response by suppressing the proliferation of lymphocytes, a type of leukocyte, which can release a variety of inflammatory factors and exacerbate the inflammatory response.

The mechanisms by which TSPCs regulate the inflammatory response can be divided into three aspects. First, TSPCs regulate the inflammatory response through the JNK and STAT3 pathways by upregulating IL-10, which in turn promotes tendon healing. Tarafder et al. [16] showed that endogenous TSPCs delivered with CTGF upregulated the expression of anti-inflammatory factors and downregulated M1 expression. IL-10 is a type of anti-inflammatory factor that has the ability to inhibit the release of proinflammatory cytokines and TSPCs upregulate IL-10 and activate the STAT3 signaling pathway to promote tendon healing. TSPCs also balance the expression of MMP3 and TIMP-3 to prevent the development of scar tissue. Second, TSPCs regulate the inflammatory response by secreting exosomes [17]. Exosomes are cell-derived vesicles that contain a variety of proteins, mRNAs, and miRNAs, are essential mediators of cell-to-cell communication and can be secreted by a variety of cells [50, 51]. Studies have shown that TSC-Exos regulate the early inflammatory response by increasing anti-inflammatory factors and inhibiting proinflammatory factors. It was shown that TSC-Exos increased IL-10 and decreased IL-6 and Cox-2. At the same time, the expression of CCR7 (a marker of M1) was significantly decreased and the level of CD 163 (a marker of M2-type anti-inflammatory macrophages) was significantly increased in the TSC-Exos group compared to other groups. Furthermore, TSC-Exos balanced the synthesis and degradation of tendon ECM by regulating the metabolic balance between MMP-9 and TIMP-1, thereby preventing the formation of scarring and fibrosis in the tendon after injury [17]. Finally, some researchers have shown that TSPCs can be induced to differentiate into vascular endothelial cells, thereby promoting tendon healing. However, TSPCs are induced to differentiate into vascular endothelial cells under specified conditions, and it is unclear whether exogenous TSPCs can stimulate neovascularisation [52]. This could be a focus for future research.

Similarly, TSPCs can be used as allografts to promote tendon repair at the site of injury. Lui et al. [53] showed that transplanting TSPCs into the site of patellar tendon injury not only reduced the number of inflammatory factors (T cells, mast cells), but also did not increase the risk of ectopic ossification. In conclusion, TSPCs play an important role in the inflammatory phase of tendon healing.

### 4.2. Tendon Stem/Progenitor Cells Promote Cell Proliferation

Tenocytes and TSPCs are the primary cell types in tendon tissue, and the proliferation and migration of tenocytes are essential for tendon repair [54]. However, tenocytes are highly differentiated cells and therefore proliferate slowly, and TSPCs and their exosomes can contribute to the proliferation of tenocytes, thereby accelerating tendon healing [55]. In addition to this, proliferation and migration of TSPCs also play a positive role in cell proliferation, according to Runesson et al. [56] who showed that the number of TSPCs increased to 40%–60% of the total cell population during the early tendon healing phase. Besides this, TSPCs can differentiate into tenocytes to increase their cell numbers.

The fact that TSPCs and their exosomes promote tenocyte proliferation is, in my opinion, significant. First, it has recently been shown that TSC-derived exosomes (TSC-Exos) secrete VEGF to regulate the proliferation of tenocytes [57]. VEGF is an important growth factor that promotes angiogenesis, collagen production, and cell proliferation. TSC-Exos-treated tenocytes not only had higher migration capacity than the control group, but also higher protein expression of colI, colIII, *α*-SMA, and Scx. Second, TSC-Exos contain large amounts of TGF-*β*, which activates the ERK1/2 signaling pathway and the TGF-*β*-Smad2/3 signaling pathway, thereby stimulating cell proliferation and migration [55]. The TGF-*β*-Smad2/3 signaling pathway plays an important role in cell proliferation and collagen production. Finally, TSC-Exos can regulate tenocyte proliferation and migration via miR-144-3p [18]. Song et al. [18] placed scaffold of photopolizable hyaluronic acid (p-HA) loaded with TDSC-Exos (pHA-TDSC-Exos) into rat Tendon-specific markers and colⅠ were found to be increased at the site of injury. In addition, TDSC-Exos showed better biomechanical properties in the treatment of tendon injuries.

TSPCs are particularly important in contributing to tendon healing as “candidate” cells in the event of tendon injury and their tendon lineage differentiation [58–60]. Although it has the capacity for spontaneous tenogenic differentiation, its ability to differentiate is significantly enhanced by growth factors or some moderate mechanical stimulation (MS) [61]. Studies have shown that a number of factors including growth factors, appropriate MS, hypoxia, and a number of genes and proteins can promote the tenogenic differentiation of TSPCs and thus improve tendon healing. Growth factors, including transforming growth factor (TGF-*β*), basic fibroblast growth factor (bFGF), hepatocyte growth factor (HGF), connective tissue growth factor (CTGF), and BMP-12, can all allow TSPCs to differentiate towards the tendon lineage [62–64].

First, Guo et al. [65] transfected adenovirus carrying the bFGF gene into human TSPCs and then transplanted FGF-2-hTDSCs into a rat injury model. Seven days after transfection, the bFGF group had higher levels of colIII production and higher expression of ScxA (which regulates the differentiation of tendon stem cells into tendon cells) compared to the control group without bFGF. The results suggest that human tendon-derived stem cells (hTSPCs) modified with the bFGF gene promoted and improved the quality of tendon healing. Further to this, Lui et al. [64] transplanted TDSC treated with CTGF and ascorbic acid into a rat patellar tendon defect model. The repaired tendons in the CTGF group not only had neatly arranged collagen fibers and increased cell distribution compared with the control group, but also reduced the risk of heterotopic ossification (HO) in this model. Similarly, BMP-12 can also induce the differentiation of stem cells to tenocytes. Xu et al. [66] applied adenoviral vectors to simultaneously transfect BMP-12 and CTGF into TDSCs, which were then transplanted to the damaged patellar tendon in rats. In vitro experiments showed that tendon marker genes, including type I and III collagen, tenascin-C, and Scx were upregulated in BMP-12 with CTGF-transfected TDSCs. In contrast, nontendon-forming marker genes were all downregulated. In vivo experiments showed that the transfected TDSCs significantly promoted patellar tendon healing.

Second, genes such as EGR1 can also promote the differentiation of tendon lines in TSPCs. Tao et al. [59] transplanted plasmids expressing EGR1 into TSPCs (EGR1-TSPCs) and found that Scx, Tnmd, and colⅠ would be highly expressed, while PPAR*γ*, RUNX2, and SOX9 were transcribed at lower levels. This result suggests that EGR1 upregulates tenogenic differentiation and inhibits adipocyte, osteoblast, and chondrocyte differentiation. In addition to this, implantation of EGR1-TSPCs into a rabbit rotator cuff model of injury showed the best therapeutic effect of EGR1-TSPCs compared to other groups.

Finally, CHIP protein has a key role in cell proliferation and differentiation, and its effect on the differentiation of TSPCs has recently been investigated. Han et al. [60] introduced CHIP-expressing lentivirus into TSPCs and observed the cell proliferation and differentiation status. The results showed that CHIIP not only increased the cell number of TSPCs, but also significantly increased the tendon-related genes Scx, Tnmd, and ColⅠ. In addition to this, implantation of TSPCs overexpressing CHIP with collagen sponges into nude mice induced a marked increase in tendon formation in vivo.

### 4.3. Tendon Stem/Progenitor Cells Stimulates Collagen Synthesis

Collagen production occurs during the cell proliferation phase when tendon fibroblasts proliferate and secrete collagen. TSPCs have been shown to promote the production of colⅠ [67]. The cell remodeling phase is the final stage of tendon healing, when the ECM undergoes remodeling to restore biomechanical function to the damaged tendon. Similarly, TSPCs exosomes can balance ECM synthesis and lectures to promote tendon healing.

The failure of biomechanical performance after tendon healing is mostly due to disordered collagen fiber arrangement and disorganized ECM composition. Primarily, TSPCs can promote tendon healing by promoting colⅠ expression. TSPCs slices transplanted into tendon defects were found to have well-arranged and longer fibrils at 4 weeks, and significantly more type I and III collagen than the control group [67]. Similarly, Tan et al. [68] established a rat patellar tendon defect model in which rat GFP-TDSCs transduced with Scx were transplanted to the injury site. Two weeks after transplantation, significantly higher colⅠ expression was found in the GFP-TDSC-Scx group in the windowed wound. This suggests that GFP-TDSC-Scx transplantation promotes early healing of tendon repair in a rat patellar tendon window injury model. Last but not least, ECM remodeling is a dynamic process accompanied by changes in matrix metalloproteinases (MMP), and MMP3 is thought to be an important enzyme in matrix remodeling [45]. During the cell remodeling phase TSPCs and their exosomes can balance the remodeling of the ECM by decreasing the expression of MMP3 and increasing the expression of TIMP-3 [45]. In addition to this, Wang et al. [45] found that TSPCs promoted colⅠ expression and increased both maximum load and ultimate stress in the repaired tendon in the TSPCs-treated group compared to the control group.

In addition to this, (Tenomodulin) Tnmd is an important transcription factor in tendon development and tendon repair. Tnmd is closely associated with the production of colⅠ and its knockdown reduces the expression of colⅠ. TSPCs can affect the synthesis of colⅠ by influencing the expression of Tnmd [69]. colⅠ is a major component of the ECM, where it is deposited and remodeled at the site of injury [70]. Its synthesis helps to promote tendon healing, but overproduction of colⅠ predisposes to the formation of scar fibrosis.

## 5. Preclinical Experiments with Tendon Stem/Progenitor Cells

TSPCs play an important role in tendon development, homeostasis, and healing. First, Scx and SOX9 are associated with the production of TSPCs during tendon development, while Tnmd regulates the proliferation of TSPCs [69]. Scx is a key transcription factor for tendon differentiation and colⅠ production. Implanting GFP-TDSC-Scx into the patellar tendon injury site of rats demonstrated that Scx increased the expression of both TSPCs Scx and colⅠ, which in turn promoted early tendon healing [68]. In conclusion, the combination of transcription factors that affect tendon development with TSPCs to promote tendon healing is a direction for future research. For example, Tao et al. [59] established a model of rotator cuff injury in the rabbit and applied TSCs and EGR1 (EGR1-TSCs) in fibrin glue carriers to the repair site. They found that EGR1-TSCs not only promoted the tendinous differentiation of TSPCs and inhibited the nontendinous differentiation of TSPCs, but also promoted rotator cuff healing. In addition to this, Mohawk (Mkx) is an important transcription factor in tendon development and differentiation that regulates the production of colⅠ [71]. Not only is the Mkx gene downregulated in tendinopathy tissues, but also tissue fibrosis and vascularity are present. Mechakra et al. [72] established an Mkx knockout model of tendon injury in mouse and found that the injured tissues underwent fibrosis and were significantly upregulated by COL3A1 and *α*-SMA. This suggests that Mkx protects tendons by inhibiting vascular fibrosis. In vitro experiments indicate that TSPCs may differentiate into myofibroblasts and hence cause vascular fibrosis, while Mkx regulates MyoD and angiogenesis. Mkx has an essential role in preventing tendon fibrosis and neovascularization.

Second, intratendinous cells and ECM work together to maintain tendon homeostasis; therefore, TSPCs and tenocytes play an important role in the biological homeostasis and regulation of tendon [36]. Although TSPCs account for a relatively low percentage, they can self-renew and differentiate into tenocytes. Finally, a variety of cytokines and growth factors promote tendon healing after tendon injury; TSPCs are one of these, but the endogenous TSPC pool may not be sufficient to recover the injury. In recent years, many investigators have taken various approaches to implant exogenous TSPCs to accelerate the tendon healing process [59]. Experiments related to the enhancement of tendon healing by TSPCs are summarized in Table 2.

## 6. Tendon Stem/Progenitor Cells May Account for the Failure of the Healing Response

As a matter of fact, while there are many beneficial aspects of TSPCs for tendon healing, it is suggested that TSPCs may also lead to a failed healing response. As we all know, not only vascularization and collagen disorders, but also chondrocytes and osteoblasts can be observed in the pathological tissue of chronic tendinopathy. Like other stem cells, TSPCs have the potential for multidirectional differentiation. During tendon healing, the differentiation of TSPCs into chondrocytes, adipocytes, and osteoblasts can lead to a “failed healing response,” which in turn contributes to the development of tendinopathy. One of the pathogenic mechanisms of tendinopathy is the misdifferentiation of TSPCs into nontenocytes [43]. Triggers of TSPCs misdifferentiation include aging, changes of ECM composition, excessive MS, and some biological active factors (inflammatory factors and cytokines), apart from drugs and metabolic diseases, which are also important contributors. As shown in Table 3.

First, the differentiation of TSPCs into chondrocytes and osteoblasts will lead to calcification of the tendon, which is one of the more common forms of tendinopathy, with a prevalence of 22% [100]. A number of factors can lead to the differentiation of TSPCs into chondrocytes and osteoblasts: repetitive mechanical loading, changes in the composition of the ECM, increases in BMP proteins, high glucose, and so forth [13, 83, 101]. For example, in the experiments of Bi et al. [13] the tendons of mice lacking the Bgn and Fmod genes underwent ectopic ossification, in addition to an increase in chondrocyte markers. The mechanism is as follows: deletion of Bgn and Fmod in TSPCs stimulates the activation of BMP-2, which increases RUNX2 expression via the Smad1-Smad5-Smad-8 pathway, thereby promoting bone formation. In response to this situation, it is crucial to inhibit the osteogenic differentiation of TSPCs for the treatment of tendinopathy.

Second, the differentiation of TSPCs into adipocytes can also impede tendon healing, such as senescence of TSPCs [77]. Studies have shown that the differentiation capacity of stem cells decreases with age, while the tenogenic ability of tendon stem cells is reduced with age [102]. Numerous nontendinous substances, such as adipocytes and osteoblasts, as well as calcification, have been detected in many animal models and human aging tendons. Aging can affect the differentiation ability of TSPCs not only directly but also by altering the condition of the niche. Meanwhile, aged TSPCs (A-TSPC) have less ability to self-renew, and A-TSPC generates more fibronectin than colⅠ [103].

Tendon marker gene expression was reduced in aging TSPCs, but lipogenic markers including PPARc2 (PPARGC1A), C/EBPa (Cebpa/CEBPA) expression was increased [104]. In addition to this, A-TSPCs express higher levels of CD44 compared to Y-TSPCs, suggesting a poorer healing capacity of the injured tissue. By contrast, Lai et al. [105] showed that the patellar tendon adipose accumulation in aged rats was not due to A-TSPC, but due to inhibition of PPAR*γ* signaling pathway by aging, thereby preventing adipogenesis in TSPCs. To verify the adipogenic capacity of senescent TSPCs, they were tested in vitro for oil red O staining, and the number of fat droplets in senescent TSPCs was found to be significantly reduced. This demonstrates that the ability of adipocytes in senescent TSPCs to be converted to adipose is reduced, which in turn leads to the accumulation of adipocytes at the injury site. PPAR*γ* signaling pathway is an essential pathway for the induction of adipogenesis. However, the PPAR*γ* signaling pathway is decreased in A-TSPC, which will prevent the transformation of adipocytes in TSPCs into adipose, leading to the accumulation of adipocytes at the injury site, which in turn impairs tendon healing.

P16 protein is a marker of aged cells and regulates the expression of genes [106]. According to a recent study, the expression of collagenⅠ and tendon-associated marker genes including Scx, Tnmd, and Bgn were reduced in A-TSPC, but the expression of P16 was significantly upregulated. Upregulation of P16 affects the tenogenic differentiation ability of TSPCs. P16 inhibits tenogenic differentiation of TSPCs by enhancing miR-217 transcription and thus decreasing EGR1 expression [77]. In response to the increased adiposity caused by A-TSPCs, methods to inhibit the adipogenic differentiation of TSPCs can be used to promote tendon regeneration. For example, VEGF has been shown to not only reduce adipocyte accumulation in tendons, but also to promote angiogenesis [107]. Understanding the mechanisms by which TSPCs induce tendinopathy can help us to develop new strategies for the treatment of tendinopathy.

## 7. TSPCs Culture and Differentiation—Hypoxic Tension, Growth Factors, Biophysical Factors

### 7.1. Hypoxic Tension

The initial development of the majority of cells is in a hypoxic state [108]. Also, it has been shown that tendon healing demands a hypoxic environment, and stem cells have a high proliferation ability under low oxygen tension [109]. Hypoxia can affect stem cell differentiation by regulating the expression of HIF-1*α*. In addition, hypoxia can increase the expression of VEGF as a way to promote angiogenesis [110].

Like other stem cells, TSPCs perform better in hypoxic conditions. Zhang and Wang [111] exposed hTSPCs to 5% oxygen and found that not only the number of TSPCs increased, but also their expression of markers of stemness was higher than in normoxia. In addition to this, tendon cell-related genes such as tenascin-C were expressed at a higher level compared to normoxia, while nontendon cell-related genes including SOX9, RUNX2 were expressed at a lower level. Low oxygen tension improves not only the proliferation capacity of normal TSPCs but also the differentiation capacity of aged stem cells [112]. Normal tendon tissue contains less oxygen and if oxygen tension is elevated, this will result in the differentiation of TSPCs into nontendon cells.

### 7.2. Growth Factor

A variety of growth factors have positive effects on the proliferation and differentiation of TSPCs, and those that have been studied include TGF-*β*, bFGF, HGF, CTGF, all of which differentiate TSPCs toward the tendon lineage [61, 64–66, 113].

Not only does TGF-*β*1 have an essential role in tendon healing, it has been shown to differentiate MSCs into tenocytes. Application of TGF-*β*1 upregulates scleral (Scx) and tendon modulating protein (Tnmd) in MSCs [114, 115]. TSPCs, as a type of MSCs, TGF-*β* is also a powerful catalyst to promote the differentiation of TSPCs into tenocytes. In a study by Guo et al. [61], the TGF-*β*1-induced group exhibited higher tendon markers, including colⅠ, Fmod, and Dcn, compared to the group that enabled spontaneous tendon differentiation. However, Tnmd was significantly lower in the TGF-*β*1 group compared to the spontaneous group, which may be due to the inhibition of Tnmd expression by factors regulated by the TGF signaling pathway.

In addition, bFGF, also known as FGF2, is a member of the fibroblast growth factor family, which has the function of promoting angiogenesis, cell proliferation, and collagen synthesis [62, 116, 117]. More importantly, bFGF can promote the differentiation of MSCs into tenocytes [118]. Guo et al. [65] transfected adenovirus carrying the bFGF gene into hTSPCs and then transplanted FGF-2-hTDSCs into a rat injury model. The results suggest that hTSPCs modified with the bFGF gene promoted and improved the quality of tendon healing.

HGF, originally found in the liver, is secreted by MSCs and can contribute to wound healing as well as activating stem cells [113, 119]. A recent investigation showed that HGF promotes TSPCs proliferation via PI3K/AKT or MAPK/ERK1/2 signaling pathways and that the number of TSPCs proliferation positively correlates with HGF concentration [63].

Apart from this, CTGF can also promote cell proliferation and differentiation, and it can also differentiate BMSCs into fibroblasts, which was found in human umbilical vein endothelial cells [120]. Lui et al. [64] transplanted TDSC treated with CTGF and ascorbic acid into a rat patellar tendon defect model. The repaired tendons in the CTGF group not only had neatly arranged collagen fibers and increased cell distribution compared with the control group, but also reduced the risk of HO in this model. Similarly, BMP-12 can also induce the differentiation of stem cells to tenocytes. Xu et al. [66] applied adenoviral vectors to simultaneously transfect BMP-12 and CTGF into TDSCs, which were then transplanted to the damaged patellar tendon in rats. In vivo experiments showed that the transfected TDSCs significantly promoted patellar tendon healing.

### 7.3. Biophysical Factors

We next review the biophysical factors that influence stem cell proliferation and differentiation, including mainly MS and the topography of the ECM [121]. It is well known that normal MS is necessary for tendon development, and MS is also considered to be one of the key factors regulating the differentiation of TSPCs [122]. Its function is to promote the proliferation and differentiation of TSPCs by upregulating the expression of mechanical growth factors [123]. For example, Popov et al. [79] observed that 8% biaxial mechanical loading increased the expression of MMPs, integrins in TSPCs. In addition, the expression of fibronectin, lumican, and versican was increased. Importantly, an increase in them not only promotes the production of collagen fibers, but also contributes to the proliferation of cells and the synthesis of ECM.

The niche of stem cells is crucial for their differentiation direction, and the niche of stem cells constituted by the topography of biomaterials can regulate the differentiation of TSPCs. Equally, the stiffness, fiber diameter, and fiber alignment of biomaterials affect the differentiation of stem cells [121]. First, matrix stiffness has a regulating effect on the differentiation of TSPCs, which is mainly via activation of the FAK-ERK1/2 signaling pathway [124]. A reduction in matrix stiffness induces chondrogenic osteogenesis, which in turn leads to tendinopathy [125]. Second, fiber diameter and fiber alignment also have an effect on the differentiation of TSPCs. Lu et al. [126] prepared silk fibroin (SF) films with different diameters and mechanical properties, and cultured rat TSPCs in 5, 10, 15, and 20 *μ*m SF films. apart from 5 *μ*m SF films, 10, 15, and 20 *μ*m SF films exhibited ultimate loads and maximum tensile forces similar to those of normal tendon. They also evaluated the morphology and viability of SF films cells and found that TSPCs in 10 *μ*m SF films exhibited oriented cell arrangement and elongated cell morphology. Moreover, the expression of tendon-related genes Scx, collagen I, and Tnmd was significantly higher in TSPCs than in other groups. These data suggest that TSPCs have the optimal biological response on 10 *μ*m SF film.

In conclusion, MS and ECM together promote the proliferation and differentiation of TSPCs Future studies are still needed to further investigate the mechanisms by which the matrix promotes stem cell differentiation.

## 8. Conclusions and Perspectives

Both acute and chronic tendon injuries (tendinopathy) are treated conservatively initially and surgically after conservative treatment has failed. Many conservative treatments including drug therapy and physiotherapy are not effective. The reason is that tendons tend to heal with scar tissue, HO, and poor mechanical properties after repair. Stem cell therapy is a new idea for the treatment of tendon injuries, especially TSPCs with spontaneous differentiation potential.

At present, the treatment of tendon injury with TSPCs has attracted a lot of attention. In particular, TSPCs are derived from a “high collagen” environment and have a better ability to proliferate in vitro compared to other stem cells [13, 127]. The TSPCs and their exosomes play an influential role in tendon repair. Tendon repair usually goes through three overlapping phases: inflammatory, proliferative, and remodeling phases [128]. During tendon healing, TSPCs and their exosomes have the following effects: anti-inflammatory, promote cell proliferation, stimulate collagen synthesis, and balance the remodeling of the ECM, respectively [16, 17, 45, 55]. Meanwhile, TSPCs were discovered to enhance collagen I synthesis. In addition, the improved biomechanical behavior of the repaired tendon was also observed [67]. However, it is necessary to further investigate the molecular mechanism of TSPCs in the treatment of tendon injury, as the mechanism of TSPCs for tendon injury is more complex. In addition, angiogenesis is a relatively vital part of tendon healing, and studies investigating the promotion of angiogenesis by TSPCs are hardly available, so this is another part of future research. In conclusion, the application of TSPCs in the management of tendon injury is a key focus of future research.

Although TSPCs play a key role in tendon healing, the negative effects of TSPCs on tendon healing cannot be ignored. It is well known that not only increased vascularity and collagen disorders, but also chondrocytes and osteoblasts can be observed in the pathological tissue of chronic tendinopathy [24]. One of the reasons for the failed healing response in chronic tendinopathy is the incorrect differentiation of TSPCs into osteoblasts, chondrocytes, and adipocytes.

HO is one of the common symptoms of chronic tendinopathy and is due to the differentiation of TSPCs into osteoblasts and chondrocytes. There is evidence that TSPCs isolated from a model of calcified tendinopathy have a higher potential for osteogenic differentiation compared to TSPCs isolated from normal tendon [43]. By contrast, both senescence and mechanical loading can cause TSPCs to differentiate into osteoblasts (chondrocytes). First, Dai et al. [101] evaluated aged rat tendons and found higher expression of the osteogenesis-related genes RUNX2, OPN, and OCN. In addition to this, the expression of BMP-2/4/7 proteins in ossifying tendons increased with age. In vitro experiments showed that TSPCs isolated from osteoclastic tendons had a high osteogenic differentiation potential. Second, Shi et al. [82] studied the effect of mechanical loading on rat TSPCs and found that RUNX2, Col1a1, and Alpl were significantly upregulated after 2% UMT stimulation for 3, 7, and 14 days. In addition to this, their study showed that the molecular mechanism of mechanical loading-induced osteogenic differentiation of TSPCs is induced through the Wnt5a-RhoA pathway.

TSPCs differentiating into adipocytes can also hinder tendon healing. For example, PGE2 at high levels (100 ng/ml) significantly inhibited the proliferation of TSPCs. Furthermore, PGE2 (100 ng/ml) upregulated the adipogenesis-related gene PPARc. In addition, high levels of PGE2 down-regulated both colⅠ and tenascin-C. An increase in PGE2 reduces cell proliferation and hinders collagen synthesis, which in turn prevents tendon healing [83]. In fact, multiple researchers have explored ways to inhibit the misdifferentiation of TSPCs. LncRNA KCNQ1OT1 can cause TSPCs to differentiate towards adipogenic osteogenesis. miR-138 can lead to downregulation of PPAR*γ*, resulting in adipogenic inhibition of human adipose tissue-derived mesenchymal stem cells [129]. Therefore, knockdown of LncRNA KCNQ1OT1 increased miR-138 expression and down-regulated PPAR*γ* and RUNX2 expression to inhibit the differentiation of TSPCs to adipocytes and osteoblasts [130].

TSPCs have an essential role in both tendon physiology and chronic tendon injury (tendinopathy), and it has promising application in tendon repair. TSPCs are an important part of maintaining tendon homeostasis, and when tendon injury is present, TSPCs should differentiate into tenocytes to promote tendon repair. Unfortunately, there are still some limitations regarding the clinically relevant nature of TSPCs. First, TSPCs are lower and scarcer in tendon tissues, so it is necessary to culture TSPCs in vitro; however, the methods of culturing stem cells have not been uniformed, resulting in cells with unsatisfactory proliferation and differentiation results. Second, TSPCs have different subpopulations, so there are not yet accurate biomarkers to track the TSPCs spectrum. A better approach would be to use genetic genealogy tracking techniques to mark TSPCs and track their lineage [39, 131]. Third, TSPCs from various sites show differences in marker expression and function, and future research should compare the similarities and differences of TSPCs from different sites [25, 132]. Finally, TSPCs for tendinopathy are not being used in the clinic, and their security needs to be examined and more studies for validation.

The future research directions are to stimulate tendon healing by activating endogenous TSPCs and to construct TSPCs niche with biological scaffolds, cytokines, and MS in order to promote the migration of autologous cells to the injury site [133]. Therefore, regarding the strategy of TSPCs for the treatment of tendinopathy, I provide the following suggestions: first, the use of exogenous TSPCs to activate endogenous TSPCs or the stimulation of endogenous TSPCs with other genes or proteins to differentiate them into tenocytes. For instance, Yu et al. [134] embedded BMSCs-exos in fibrin and injected it into the defective patellar tendon of rats. They found that BMSCs-exos not only promoted the proliferation of endogenous TSPCs but also promoted the expression of colⅠ and Mkx, tenomodulin, which in turn promoted tendon healing. Second, cytokines or growth factors are used in combination with TSPCs to enhance the tendon differentiation of TSPCs. Distinct growth factors have different biological effects during tendon healing. TGF-*β*1, insulin-like growth factor-1 and growth and differentiation factor-5 were added as supplements to TSPCs, and an increase in the expression of colⅠ and tendon-related genes was found in TSPCs [135]. Third, to create a suitable niche for the tendon lineage differentiation of TSPCs, such as ECM combined with hydrogel to promote the sustained generation of TSPCs. Ge et al. [136] injected TSPCs-Gel into rat injured Achilles tendons using DNA hydrogel as an excellent artificial ECM for proliferation and protection of TSPCs (TSPCs-Gel). They found that TSPCs-Gel injection not only promoted the healing of rat tendon, but also improved its ultimate loading ability. Finally, TSPCs were used as seed cells to find suitable scaffolds that could both inhibit misdifferentiation of TSPCs and promote tendon differentiation of TSPCs. Xu et al. [137] evaluated the utility of TDSCs in poly(L-propylidene-*co*-*ε*-caprolactone)/collagen (P(LLA-CL)/Col) scaffolds for the regeneration of rabbit patellar tendon defects under MS. They found that TDSCs-P(LLA-CL)/Col constructs could significantly promote the repair of injured rabbit patellar tendons by enhancing collagen production and expression of tendon-related proteins. In addition to this, the combination of TSPCs with genes and proteins for the therapeutic treatment of tendon injury is also a focus of future investigation. Kang et al. [138] established that TDSCs were infected with recombinant Lrrc32 overexpressing lentivirus (LV-Lrrc32) and then locally injected into the injury site of rats, and the results showed that Lrrc32 promoted the tendon differentiation of TDSCs in vivo and facilitated the healing of tendons in rats. In conclusion, TSPCs deserve further investigation as a potential cell therapy, both in terms of the factors that induce its multidirectional differentiation and the mechanisms by which it promotes tendon healing.

## Figures and Tables

**Table 1:** Summary of general comparisons between tenocytes and TSPCs.

	TSPCs	Tenocytes
Morphology	Pebble-shaped, spindle-shaped, and rounded	Spindle-shaped (large)
Rate of proliferation	Relatively fast	Slower
Potential for differentiation	TSCs have the ability to differentiate into tenocytes as well as into several nontendon cell types including adipocytes, chondrocytes, and osteocytes	Mature tenocytes without the ability of differentiation, but investigators claim that tenocytes have some ability to differentiate into chondrocytes
Markers	Scx, Tnmd, and type I collagen	Oct-4, SSEA-4, and nucleostemin, CD44, CD90, and CD105, Scx, Tnmd, and tenascin-C, and type I collagen
Colonies formed	Dense and close	Large and sparse

**Table 2:** Summary of results and characteristics of the studies which investigated the effects of TSPCs in tendon healing.

Animal type	Models establish	Dosage	Time postoperation	Outcome	Conclusion	Reference
GFP-positive transgenic male rats	Achille tenotomy and repair	GFP-TSC sheet	2, 4 weeks	Round shaped cells↑ cell numbers, ECM↑ Tnmd, and type I collagen↑ Ultimate Strength↑	Better cellular alignment, elongation, and densely aligned collagen arrangement in the regenerated tissue at the TSC sheet grafted tendon defect area was observed	[67]
Non-GFP SD male rats	Patellar tendon window injury	GFP-TDSC, CTGF ascorbic acid	2, 4, 8, 16 weeks	Ultimate stress↑ collagen fibers↑ spindle-shaped cells↑ ectopic mineralized tissues↓	The transplantation of TDSCs promoted tendon repair up to week 16	[64]
Young adult horses	Flexor tendinitis induced by collagenase	TSPCs: 5 × 10^6^ TSPCs in 0.15 ml of sterile phosphate-buffered saline	1, 2, 4, 6, 12 weeks	Type I collagen, COMP, and tenomodulin mRNA↑ maximum stresses↑ collagen fibers were significantly more aligned	Treatment of collagenase-induced flexor tendon injury with TSPCs not only improved the tensile strength of the repaired tissue, but also improved collagen fiber alignment	[73]
SD male rats	Window wound in the patellar tendon and repair	NA	3, 7, 14 days 4, 6, 8 weeks	CD44+, Sca+↑ Scx, Tnmd, smad8↑ Oct4+, Nanog+, SOX2+, nucleostemin+ CD146↑	The LRCs participated in tendon repair after injury via migration, proliferation, activation for tenogenesis, and increased pluripotency in the window wound	[15]
SD rats	Patellar tendon window injury	GFP-TDSC fibrin	1, 2, 4, 8, 16 weeks	Fiber arrangement ↑CD3, CD68 (1, 2 weeks) ↓CD163↓ Vascular distribution↓ transplanted cells↓	Transplantation of allogeneic TSPCs to patellar tendon injury sites in rats not only promotes tendon healing, but also shows a weak immune response	[53]
SD rats	PT transection and repair	NA	2 days 1, 2 weeks	CD 146^+^TSC ↑TIMP-3↑ IL-10↑ IL-6↓	CTGF delivery improves the quality of tendon healing by activating TSPCs. Similarly, TSPCs have an anti-inflammatory effect	[16]
Female rabbits	Rotator cuff tendon defect	Cell-seeded scaffold (knitted silk—collagen sponge + collagen gel containing allogenous rTSPCs)	4, 8, 12 weeks	Collagen I, collagen III, Bgn, and TNC ↑ fibroblastic cells↑ stiffness, maximum force, emerge, modulus, and stress at failure↑	Allogenous TSPC-seeded scaffolds can promote healing of rotator cuff injury	[49]
SD rats	Achilles tendon defect	Seeded with TSPCs (ECM + TSPCs group, *N* = 12, 5 × 10^5^ cells per scaffold)	4 weeks	Organized collagen fiber structures↑ fibroblast-like cells↑ collagen type I ↑stiffness↑	The tendon ECM scaffold inoculated with TSPCs not only promotes cell proliferation and stimulates collagen synthesis, but also improves the mechanical properties of the healing tendon	[74]
Outbred non-GFP SD male rats	Patellar tendon Window injury	NA	2, 4, 8 weeks	mRNA expression of Scx ↑Swelling of the knee↓ degree of inflammation↓ fiber arrangement↑ ultimate stress↑ collagen type I↑	GFP-TDSC-Scx group might promote early tendon repair by increasing the expression of collagen type I in the window wound	[68]
Male outbred SD rats	ACL excision and reconstruction	GFP-TDSC sheet	2, 6, 12 weeks	Cell alignment ↑collagen birefringence↑ ultimate load↑	The TDSC sheet improved early graft healing after ACL reconstruction in the rat model	[75]
SD rats	Achilles tendon rupture and repair	CM (HGF + TSCs)	2 weeks	MMP-2, MMP-9↑ VEGF↑ Biomechanical properties↑	CM treatment promotes repair and functional recovery of Achilles tendon ruptures	[76]

*Note:* ↑, significant increase; ↓, significant decrease. NA, not applicable; TSPCs, human tendon stem/progenitor cells; Bgn, biglycan; CTGF, connective tissue growth factor; TNC, tenascin-C; COMP, cartilage oligomeric matrix protein; LRCs, label-retaining cells; Tnmd, tenomodulin; Scx, scleraxis; ECM, extracellular matrix; ACL, anterior cruciate ligament; MMP-2, matrix metallopeptidase 2; MMP-9, matrix metallopeptidase 2; VEGF, vascular endothelial growth factor, HGF, hepatocyte growth factor.

**Table 3:** Factors that induce nontendon lineage differentiation of TSPCs.

Factors	Stem cell source	Outcomes	Conclusion	Reference
P16 (gene)	Human	Inhibits tenogenic differentiation of TSPCs	Aging marker gene P16 via microRNA signaling pathways	[77]
Extracellular matrix (ECM)	Mouse	Osteogenic differentiation	Double knockout of ECM with biglycan and fibronectin induces differentiation of TSPC into osteoblasts and a reduction in collagen Ⅰ	[13]
Mechanical loading	Sprague-Dawley rats	BMP-2 mRNA, ALP↑	Promoting osteogenic differentiation by upregulating BMP-2 expression following repetitive stretch loading	[78]
Mechanical stimulation (8% double axis MS)	Human	Fibromodulin, lumican, and versican↑ Collagen I↑ MMP9, 13, and 14↑	Mechanical stimuli are mediated through ERK1/2 and p38, which were significantly activated in 8% biaxial-loaded TSPC	[79]
Mechanical stimulation (8% MS)	(SD) rats	Adipogenic, osteogenic, and chondrogenic differentiation SOX9, CollagenⅡ↑	Mechanical loading activates mTOR signaling in TSPCs and results in their differentiation by nontendon lineage	[80]
Uniaxial mechanical stimulation (8% MS)	Rats	Osteogenic differentiation RUNX2, Dlx5, Alpl, and Collagen I↑ Wnt5a, Wnt5b, and P-JNK protein↑	UMT induced the osteogenic differentiation of rTDSCs via the Wnt5a/Wnt5b/JNK signaling pathway	[81]
Uniaxial Mechanical stimulation (2% MS)	Rats	Osteogenic differentiation RUNX2 mRNA, Collagen I, Alpl mRNA↑ Wnt5a↑	UMT-induced osteogenic differentiation of rTDSCs via the Wnt5a-RhoA pathway, which might contribute to ectopic ossification in tendon tissue due to mechanical loading	[82]
PGE2	Rats	Adipogenic and osteogenic differentiation TSPC proliferation↓	TSPCs are induced by PGE2 to differentiate into adipocytes and osteocytes, which in turn lead to adipose accumulation and calcification in tendinopathy	[83]
PGE2 (0–10 ng/ml)	Rabbit patellar	Adipogenic and osteogenic differentiation	PGE2 not only prevents the proliferation of TPSCs, but also induces it to differentiate into adipocytes and osteoblasts, which in turn leads to degenerative changes in the tendon	[84]
IL-6	SD female rat	Scx, Tnmd, Egr-1↓ Fmod, Lum, Collagen I, Collagen Ⅲ↓ cell proliferation↑	IL-6 stimulates TPSCs proliferation but inhibits their tenogenic differentiation	[85]
IL-10	SD rats	Collagen I, CollagenⅢ↓ Scx, Tnmd↓ Egr-1↓ Cell proliferation of TSPCs↑	IL-10 promoted the proliferation and migration of TDSCs, but also inhibited its tenogenic differentiation	[86]
IL-1*β*	CD-1 mouse	Egr-1, Collagen I, Collagen Ⅲ↓ Mkx, MMP13↑	IL-1*β* irreversibly inhibits tenogenic differentiation of inTPCs	[87]
BMP-2	SD rats	GAG, Acan↑Dcn, Bgn, Fmod ↓	BMP-2 promoted osteogenic, adipogenic, and chondrogenic differentiation but inhibited tenogenic marker expression of TSPCs	[88]
CTRP3	Mouse	PG↑, Col2a1, Acan, Fn1, and SOX9↑ Scx, Mkx, Tnmd↓	CTRP3 is significantly increased in loading tendinopathy and it stimulates chondrogenesis and inhibits tenocyte production	[89]
High glucose	Rats	Scx, Tnmd, Collagen I↓ cell proliferation↓	High glucose could inhibit proliferation, induce cell apoptosis, and suppress the tendon-related markers expression of TDSCs in vitro	[90]
Nesfatin-1	(SD) rats	Osteogenic differentiation Scx, Mkx, Tnmd↓ Collagen I, ALP, and RUNX2 ↑	Nesfatin-1 accelerated the pathogenesis of HO through the mTOR pathway in vivo	[91]
Dex	Human patellar tendon	Collagen I↓ PPAR*γ*, SOX9↑	Higher concentrations of Dex treatment induced hTSPC differentiation into nontenocytes (adipocytes and chondrocytes)	[92]
Dexamethasone	(SD) rats	Collagen I and Tnmd mRNA↓ Scx mRNA↓	Dexamethasone inhibits the differentiation of TSPCs to tenocytes by inhibiting the Scx gene	[93]
Celecoxib	Male C57 mouse	Scx, Egr1 mRNA↓ Tnmd, Bgn, Dcn, Fmod, Tnc, and Eln mRNA↓ cell number n.a	Celecoxib inhibits tenocytic differentiation of TSPCs but has no effects on cell proliferation	[94]
Diabetes mellitus (DM)	(SD) rats	Osteogenic and chondrogenic differentiation OPN, OCN, SOX9, Collagen Ⅱ↑ BMP-2, ALP↑ collagen Ⅰ, Tnmd↓	TPSCs in diabetic rats not only have reduced ability to proliferate, but also have higher osteochondral formation and lower tenogenic differentiation	[95]
Cholesterol	Rats	LC3-II↑, ROS↑ CAT, NOX4↑	High cholesterol induces apoptosis by up-regulating ROS in TPSCs to activate the FOXO1 pathway, thereby causing tendinopathy	[96]
MiR124	Human	Collagen Ⅰ, Ⅱ↓ Fmod, Dcn↓	MiR124 hampers collagen production in TSPCs by inhibiting the expression of Egr	[97]
Wnt/*β*-catenin signaling	(SD) rats	Scx, Mkx, and Tnmd↓ Bglap, Alpl↑	Wnt/*β*-catenin signaling is a repressor for tenogenic gene expressions	[98]
ERK1/2 signaling pathway (normoxic (20% O2) conditions)	Rats	ALP activity↑ gene (ALP, osteocalcin, collagen I, and RUNX2) ↑	ERK1/2 signaling pathway is involved in the osteogenic differentiation of TSPCs under normoxic conditions	[99]

*Note:* ↑, significant increase; ↓, significant decrease. n.a, not affected; TSPCs, tendon stem/progenitor cells; MMP-9, matrix metalloproteinase-9; MMP-13, matrix metalloproteinase-13; MMP-14, matrix metalloproteinase-14; SOX9, SRY-box9; rTDSCs, rat t tendon-derived stem cells; PGE2, prostaglandin E2; IL-6, interleukin-6; IL-10, interleukin-10; Scx, scleraxis; Tnmd, tendmodulin; Mkx, Mohawk; HO, heterotopic ossification; ALP, alkaline phosphatase; RUNX2 ↑, Runt-related transcription factor 2; OPN, osteopontin; OCN, osteocalcin; BMP-2, bone morphogenetic protein 2; Dcn, decorin; Acan, aggrecan; Fmod, fibromodulin; GAG, glycoaminoglycans; Alpl, alkaline phosphatase.

## Data Availability

Data sharing is not applicable to this article as no new data was created or analyzed in this study.
